# The effects of B cell depletion on immune related adverse events associated with immune checkpoint inhibition

**DOI:** 10.1186/s40164-020-00167-1

**Published:** 2020-05-25

**Authors:** Rasmus Strøm Risbjerg, Mie Vennize Hansen, Anne Sofie Sørensen, Tue Wenzel Kragstrup

**Affiliations:** 1 Department of Biomedicine, Aarhus University, Skou Building, 8000 Aarhus C, Denmark; 2 Department of Rheumatology, Aarhus University Hospital, Aarhus, Denmark

**Keywords:** B cell depletion, Immune checkpoint inhibition, Immune related adverse events, Immune mediated inflammatory disease, Hypothyroidism

## Abstract

This letter describes the potential effect of B cell depletion on immune related adverse events associated with immune checkpoint inhibition. B cell depleting agents such as rituximab reduce B cell to plasma cell differentiation and antibody production. This treatment strategy is used in several immune mediated inflammatory diseases such as rheumatoid arthritis and small vessel vasculitis. The immune related adverse events associated with immune checkpoint inhibition resemble immune mediated inflammatory diseases. Here, we report a lower incidence of hypothyroidism in a trial of combined B cell depletion and immune checkpoint inhibitor treatment compared with studies of immune checkpoint inhibitor monotherapy. This letter aims to increase awareness of the immune related adverse events associated with immune checkpoint inhibition in future clinical trials of immune checkpoint inhibition together with B cell depletion (primarily trials of B cell lymphomas). Hopefully, observations from these clinical trials can guide future treatment strategies to treat or prevent immune related adverse events associated with immune checkpoint inhibition.

The next decade will bring fundamental knowledge about the immunology behind the immune related adverse events (irAEs) associated with immune checkpoint inhibition (ICI). This knowledge will come as a consequence of new clinical trials of ICI together with B cell depletion. These treatment regimens are designed to improve outcomes for patients with B cell hematologic malignancies. However, reports of the irAE profile will be a major add-on benefit from these clinical trials.

During ICI 35% of cancer patients develop irAEs. This type of adverse events resembles immune mediated inflammatory diseases (IMIDs). When treating cancer, we try to induce immunological reactions against the tumor cells. When treating IMIDs, we try to dampen immunological reactions causing inflammation. Immune modulating therapy can therefore be a double-edged sword. However, the two processes might not be mutually exclusive.

ICI refers to blocking antibodies targeting the programmed cell death protein 1 (PD1) and cytotoxic T-lymphocyte-associated protein 4 (CTLA4) pathways. PD1 and CTLA4 are negative regulators of T cell activity and are upregulated in many malignancies. In this way, cancer cells evade immune surveillance. ICI results in increased activity of the immune system and increased recognition and elimination of cancer cells. Therefore, ICI is emerging as a potential treatment option for many malignancies including lymphoma. B cell depleting agents bind to B cell specific membrane proteins causing complement-dependent cytotoxicity and antibody-dependent cellular cytotoxicity. E.g. rituximab binds to CD20 found on mature B cells and is routinely used in the treatment of B cell lymphomas. This results in depletion of the cancerous B cells expressing the CD20 protein on their surface. However, depletion of all mature B cells will also reduce antigen presentation, B cell to plasma cell differentiation and antibody production. Therefore, rituximab is also used in the treatment of several IMIDs such as rheumatoid arthritis and small vessel vasculitis.

Little is known about the immunological reactions causing irAEs. Some irAEs are associated with the presence of autoantibodies implying a role for B cells. The most frequent of these autoantibody-associated irAEs is thyroiditis.

Until now, only one clinical trial combining ICI with B cell depletion has been completed. 2 To investigate whether rituximab alters the frequency of irAEs we compared the incidence of hypothyroidism in this phase II trial with the incidence of hypothyroidism in comparable studies of PD1 inhibitor monotherapy.

The completed phase II clinical trial was a study of 32 patients with rituximab-sensitive follicular lymphoma treated with pidilizumab and rituximab [1]. Pidilizumab was administered at 3 mg/kg intravenously every 4 weeks for 4 infusions. Furthermore, eight optional infusions every 4 weeks were available for patients with stable disease or tumor regression. Rituximab infusions were administered at 375 mg/m^2^ intravenously 17 days after the first infusion of pidilizumab and weekly for 4 weeks. Of the 32 patients, two were ineligible and not treated. Thirty patients were available for toxicity analysis, but only 29 received the therapy. The incidence of hypothyroidism in the 29 patients that received combination therapy was 0% (confirmed by personal correspondence with the authors).

This incidence contrasts with larger studies with PD1 inhibition monotherapy reporting between 6% and 10% of patients experiencing hypothyroidism (Table 1) [2–4] The studies in Table 1 were selected to best match the phase II combination therapy trial in regard to PD1 monotherapy and follow-up time. Therefore, the inclusion criteria were (1) treatment with PD1 inhibitor monotherapy, (2) follow-up time of around 15 weeks, (3) more than 200 patients included and toxicity result available for hypothyroidism.

**Table 1:** Characteristics and adverse events from ICI monotherapy studies

Study	Patients	Therapy (dose)	Hypothyroid events of any grade (%)	Hypothyroid events of grade 3–5	Median follow-up (range)
Westin [1]	29	Pidilizumab (3 mg/kg)	0 (0)	0	15.4 months
Weber [2]	452	Nivolumab (3 mg/kg)	49 (10.8)	1	19.5 months/13 months^a^
Bellmunt [3]	266	Pembrolizumab	17 (6.4)^b^	0	14.1 months
Herbst [4]	339	Pembrolizumab (2 mg/kg)	28 (8.3)	0	13.1 months (8.6–17–7)

^a^Adverse events were reported up to 30 days after last treatment and treatment duration was up to 12 months at a maximum, for that reason follow-up time regarding adverse events can add up to no more than 13 months

^b^10 patients (3.8%) experienced hyperthyroidism

The general purpose of ICI is to induce immunological reactions against the tumor cells. Therefore, any treatment of irAEs will potentially compromise the anti-tumor response. It is not known whether concurrent use of B cell depletion therapy reduces the efficacy of immunotherapy in non-B cell lymphoma malignancies.

Obviously, the small sample size of the completed phase II clinical trial (29 patients) is a major limitation. Thyroiditis was chosen because it is one of the most frequent irAE. We did not compare any of the other irAEs. This letter primarily aims to increase awareness of the irAEs associated with ICI in future clinical trials of ICI together with B cell depletion. A list of these ongoing trials and their completion dates can be seen in Table 2. The ongoing trials were found on clinicaltrials.gov by searching for ‘rituximab’, ‘obinutuzumab’ or ‘ofatumumab’ together with ‘pidilizumab’, ‘nivolumab’ or ‘pembrolizumab’ including only clinical trials with a treatment arm combining CD20 targeted antibody and PD1 inhibition. Hopefully, observations from these clinical trials can guide future treatment strategies to treat or prevent irEAs associated with ICI.

**Table 2:** List of ongoing PD-1 inhibition and rituximab combination therapy studies

Study (NCT)	Phase	Patients	ICI	Study start	Study completion	Conditions treated
NCT02541565	1	33	Pembrolizumab	November 2015	December 18, 2018^a^	DLBCL, FL
NCT03259529	1/2	30	Nivolumab	March 2017	December 27, 2020	DLBCL
NCT03749018	2	30	Nivolumab	January 2019	December 31, 2021	B-cell non-Hodgkin lymphoma
NCT03704714	1/2	30	Nivolumab	November 2018	June 11, 2022	DLBCL
NCT03719131	2	44	Nivolumab	June 2019	October 31, 2022	Stage III-IV melanoma
NCT03630042	2	42	Pembrolizumab	August 2019	April, 2023	Waldenström’s macrogloulinamia
NCT03934814	1	88	Pembrolizumab	May 2019	September 23, 2023	Solid tumors, DLBCL, indolent lymphoma
NCT02677155	2	20	Pembrolizumab	January 2016	January, 2024	FL
NCT03245021	1	39	Nivolumab	September 2017	June, 2024	FL
NCT03995147	2	51	Pembrolizumab	August 2019	August 20, 2024	DLBCL
NCT03121677	1	20	Nivolumab	October 2018	January 31, 2028	FL

*DLBCL* diffuse large B-cell lymphoma, *FL* follicular lymphoma

^a^No results posted

## Data Availability

Not applicable.

## Abbreviations

CTLA4: Cytotoxic T-lymphocyte-associated protein 4
ICI: Immune checkpoint inhibition
IMID: Immune mediated inflammatory disease
irAE: Immune related adverse events
PD1: Programmed cell death protein 1
